# Association of infant growth with emergence of permanent dentition among 12 year-aged southern Chinese school children

**DOI:** 10.1186/s12903-019-0737-1

**Published:** 2019-03-13

**Authors:** Hai Ming Wong, Si-Min Peng, Colman P. J. McGrath

**Affiliations:** 1 Paediatric Dentistry & Orthodontics, Faculty of Dentistry, The University of Hong Kong, Hong Kong SAR, China; 2 Dental Public Health, Faculty of Dentistry, The University of Hong Kong, Hong Kong SAR, China

**Keywords:** Tooth emergence, Growth trajectory, Growth rate

## Abstract

**Background:**

There is a need to comprehensively investigate the relationship between tooth eruption and infant growth to explain the theory of tooth emergence. This study aimed to investigate the association between infant growth during the first year of life and the emergence of the permanent teeth.

**Methods:**

A random sample of 668, 12-year-old students was recruited from a birth cohort. Erupted permanent tooth number was recorded. The association of infant growth (growth trajectories and growth rates) and permanent tooth emergence was examined through logistic regression analyses. The regression model was adjusted by potential confounders including gender, gestational age, mode of delivery, type of feeding, parental education, and health status.

**Results:**

The response rate was 76.9% (*n* = 514). Two hundred and forty-five (47.7%) children had all 28 permanent teeth erupted. Infants who had higher birth weight *z*-scores and those who had grown slowly during the first three months of life were more likely to have complete permanent teeth emergence at their 12-year-old in both unadjusted (*p* <  0.01) and adjusted model (adjusted for gender, gestational age, mode of delivery, type of feeding, parental education, and health status, *p* <  0.01). However, no significant association was found between the growth trajectories and permanent tooth emergence in either unadjusted or adjusted models (*p* > 0.05).

**Conclusion:**

Birth weight and infant growth during the first three months of life might be associated with permanent tooth emergence at their 12 years of age. This association may be applied in the assessment of risk for dental caries or malocclusion.

## Background

Tooth eruption is the process of a tooth moving from its site developed in alveolar bone to its functional position in occlusion [1]. The eruptive movements are motivated by the root formation and include five stages: preeruptive movements, intraosseous eruption, mucosal penetration, preocclusal eruption, and postocclusal eruption [2]. Tooth emergence is part of the eruption process which a tooth penetrates into the oral cavity from within its follicle in the alveolar process and mucosa of the maxilla or mandible [3].

Disturbances in timing or sequence of eruption may result in a chain of complications such as malocclusion, periodontal disease, and dental caries; and subsequently increase the associated dental and orthodontic treatment needs. Furthermore, the consequence extends beyond simply ‘oral health’ as it also has implications for child development (both physical and psychological) and general health status [4, 5]. Although a combination of genetic and environmental factors is mentioned [6], the mechanism responsible for tooth eruption/emergence remains uncertain. Therefore, the possible explanation of the theory of tooth emergence is one of the ‘hot spots’ in dental research globally.

Early life programming is thought to be of great importance in later life health outcomes. In particular, it is accepted that early childhood events, especially in the first year of life, can have considerable impact not only on childhood survival but also morbidities of later life [7]. Moreover, life course epidemiological studies of oral health are gradually recognised as important to identify pathways of oral health, so as to inform best practice of oral health care services for children [8]. Thus, the first year of life is identified as a ‘critical period’ [7] for general and/or oral health.

The hypothesis of early life programming is supported by epidemiological evidence in humans revealing that rapid infant growth increases the risk of metabolic syndrome [9], earlier pubertal onset [10], higher childhood body mass index [11], coronary heart disease [12], asthma [13], stroke [14], hypertension, and Type 2 diabetes [15]; while slow infant growth increased the susceptibility to non-infectious illness [16, 17]. Research work in dental fields has mainly focused on the influence of body weight on primary tooth eruption and dental caries [18, 19]. As the development of permanent teeth (except for third molars) initiates from 3.5–4 months (first molars) in utero to 8.5–9 months of age (second molars), the nutrition status in terms of changes of body weight during this period may have influence on the timing of tooth eruption [20]. Although some researchers tried to investigate the relationship of childhood growth and subsequent dental caries experience, or tooth eruption, no conclusion can be provided as for a large part of these studies are of cross-sectional design. To our knowledge, no study has been carried out to explore the association between infant growth and the permanent tooth emergence when the subjects reached 12 years old. Thus, the current study was designed to investigate if variations of growth during the first year of life had an influence on the emergence of permanent teeth at 12 years old in a sub-sample of a Chinese birth cohort.

## Methods

### Study population

The oral health survey was of a cross-sectional design which fused into a longitudinal Chinese birth cohort study. The participants were randomly selected from a longitudinal Chinese birth cohort ‘Children of 1997’. Eighty eight percent of all infants that were born between April 1st and May 31st 1997 in Hong Kong were recruited in the cohort (*N* = 8327) [21]. After 13 years’ follow-up, there were 7381 children remained in the cohort [22]. It was estimated that a sample of 470 students would have an 80% statistical power of detecting an odds ratio (OR) of 1.50 in the chance of having complete permanent tooth emergence (estimated at approximately 80%) with 1 unit raises in birth weight z-score, together considering a design effect of cluster sampling and level of significance (alpha) set at 0.05. However, to compensate for possible non-participation, the study sample was increased by 25% to 650 students. All local secondary schools in Hong Kong were the primary sampling units (by law all students are required to attend secondary school). A sample of approximately 10% of all local secondary schools (45 schools) from 18 districts in Hong Kong was selected randomly. Participants of the ‘Children of 1997’ birth cohort were invited to take part in the study within each school. Written consent was obtained from all participants’ parents or legal guardians before the oral examination and verbal assent was obtained from all participants on the examination day. In order to assure confidentiality, a de-identified data file was created after the oral health survey data were merged with medical records at Maternal and Child Health Centers (MCHCs). The protocol for this study was reviewed and approved by the Institutional Review Board of the University of Hong Kong/Hospital Authority Hong Kong West Cluster (reference number: UW 12–140).

### Data collection

#### Assessment of tooth emergence status

The clinical examination was conducted in the student’s school within two months before the participant’s 13th birthday. In order to gain adequate visibility during examination, the students were asked to rinse their mouth before lying supine on portable dental chairs, and then their gingivae were cleaned with gauze to eliminate food debris for proper visibility through examination. Prior to the field survey, two dentists who were blind to the students’ information and the objectives of the study were trained and calibrated by an experienced Paedodontist (HMW) in clinical assessment of the emergence of a tooth. In the field study, dental examinations were performed using a plain intra-oral disposable mouth mirror with a built in light-emitting diode (LED) light source and a blunt probe. The probe was used to sense or prove the occurrence of newly erupted teeth. The diagnostic criterion for an emerged tooth was when any portion of the crown has perforated the oral mucosa and was visible through the oral mucosa [3, 23]. Records on the status of emergence of the 28 permanent teeth (two incisors, one canine, two premolars, and two molars in each quadrant of the mouth) were filled in the World Health Organization (WHO) oral health assessment form [24]. Hypodontia and extractions were unavailable to assess as this study was conducted in the field where no radiographic equipment or dental history was available.

Even though the diagnostic criteria for the emergence of teeth have been clearly stated and discussed prior to the survey, it could still be difficult to determine whether a tooth has emerged or not when it was close to emergence or has just barely emerged. To guarantee the consistence throughout the study, blind duplicate oral exam was conducted among 10% participants who were randomly selected from the study sample to examine the intra- and inter-examiner reliabilities.

#### Infant growth and development information

The participants’ information on their growth and development was retrieved from the well documented medical records at MCHCs. The data of the ‘Children of 1997’ birth cohort included body weights at birth, and 1, 3 and 12 months of age; information on birth characteristics (gestational age, mode of delivery, and birth-associated congenital conditions, type of hospital), and type of feeding. Five gender-specific growth trajectories were generated according to body weight at different time points (at birth, 1, 3 and 12 months of age) through latent class analysis in the whole cohort through SAS (version 9.1) [22]. Firstly, weights were interpolated to exact ages by linear interpolation using PROC EXPAND procedure. Secondly, maximum-likelihood hierarchical cluster analysis for mixtures of spherical multivariate normal distributions was carried out for those individuals with complete weight measures at 4 time points (at birth, 1, 3 and 12 months of age). Thirdly, the probabilities of each individuals belonging to each trajectory were determined [22]. The five trajectories were: Trajectory I had a lower-to-average birth weight and a decelerated growth rate; Trajectory II had a lower-to-average birth weight and a mean growth rate; Trajectory III had a birth weight similar to the WHO average and an accelerated growth rate; Trajectory IV had a birth weight similar to the WHO average and a mean growth rate; and those in Trajectory V had a higher-to average birth weight and an accelerated growth rate [25]. Each student was categorized into one trajectory group exclusively. Growth rates from birth to 3 months of age and 3 to 12 months of age were divided based on the differences on weight z-score. Z-score equals to the difference of the individual weight and median weight of the population divided by the standard deviation of the population.

#### Socio-demographic status

Socio-demographic status, with regards to the child’s gender and parental educational attainment, was also retrieved from the medical records at MCHCs.

### Statistical analysis

Data of tooth emergence status from the oral health survey at 12 years old were combined with data from ‘Children of 1997’ in terms of birth characteristics, body weight during the first year of life, growth trajectories, and socio-demographic status. Descriptive statistics were used to summarise demographic data in terms of birth characteristics, socio-demographics and permanent tooth emergence through t-test or ANOVA for continuous data, and χ2 test for categorical variables. Included participants were those with growth trajectories. Those without data on growth trajectories were excluded. Variance on demographic data between the included and the excluded students were compared.

The association of infant growth with permanent tooth emergence at 12 years of age was examined through logistic regression models. The permanent tooth emergence was the dependent variable, with code 1 as the complete emergence group and 0 as the incomplete emergence group. The main independent variables were growth variables- growth trajectories and growth rates (from birth to 3 months of age, and from 3 to 12 months of age). We put these two growth variables in the model separately. The co-variables in the study, obtained from MCHCs were: (a) birth characteristics: gestational age (37, 38, 39, 40, or ≥ 41 weeks) and mode of delivery (natural labour, assisted natural labour, cesarean birth); (b) health status (presence or absence of congenital conditions; (c) highest parental educational attainment (≤ 9th grade, 10th – 11th grade, or ≥ 12th grade); (d) type of feeding (never breastfed, exclusively breastfed 3 months or partially breastfed, exclusively breastfed for 3 months or more), and (e) student’s gender. Furthermore, models were additionally adjusted for birth weight z-score so as to differentiate the growth effect irrespective of the birth weight. The OR was reported with 95% confidence intervals (CI).

Further analyses at the tooth level were performed if significant differences were found in logistic regression analyses at the subject level. Statistical analysis was performed using IBM SPSS Statistics 20.0 (SPSS Inc., Chicago, Illinois, USA).

## Results

The response rate was 76.9% as 514 written consents were obtained from 668 parents. Four hundred and eighty-five of those 514 students had the complete profile on birth characteristics (Table 1). Among the 485 students, there were 241 boys and 244 girls. Included participants had a higher weight-for-age z-score at birth, 3 months and 12 months than the excluded (*p* <  0.05). Children included in the study had higher proportion of natural birth and less proportion of caesarean birth (p <  0.05). No significant difference was found in other information in terms of gender, health status at birth, type of feeding, parental education attainment, or oral health data among participants and non-participants (*p* > 0.05) (Table 2).

**Table 1:** Baseline Characteristics by trajectory and growth Rrate for 485 children in the Hong Kong “Children of 1997” birth cohort

Characteristics	Trajectory					Mean Growth Rate (SD)	
	I	II	III	IV	V	0–3	3–12
	(*n* = 81)	(*n* = 114)	(*n* = 102)	(*n* = 100)	(*n* = 88)	Months	Months
Erupted permanent tooth number; mean (SD)	26.3(2.0)	25.8(3.0)	26.3(2.7)	26.4(2.4)	26.6(1.9)		
Complete emergence^a^							
Yes	43.2	43.0	54.9	47.0	51.1	0.11 (0.91)*	0.02 (0.77)
No	56.8	57.0	45.1	53.0	48.9	0.37 (0.85)*	− 0.08 (0.64)
Gender^a^							
Female	61.7	51.8	45.1	52.0	42.0	0.27 (0.89)	0.03 (0.63)
Male	38.3	48.2	54.9	48.0	58.0	0.33 (0.95)	−0.04 (0.79)
Weight for age z-score; mean (SD)							
Birth*	−0.70 (0.99)	− 0.66 (0.75)	− 0.19 (0.64)	0.24 (0.63)	0.51 (0.90)		
3 months*	−0.92 (0.57)	−0.40 (0.38)	0.40 (0.67)	0.31 (0.40)	1.06 (0.64)		
12 months*	−1.14 (0.50)	−0.31 (0.44)	0.52 (0.33)	0.02 (0.35)	1.19 (0.67)		
Gestational age (weeks)^a^							
37*	14.8	9.6	11.8	2.0	1.1	0.83 (0.98)	0.04 (0.70)
38*	19.8	27.2	25.5	15.0	22.7	0.38 (1.00)	0.14 (0.79)
39*	38.3	29.8	29.4	29.0	23.9	0.21 (0.82)	−0.05 (0.65)
40*	14.8	26.3	24.5	34.0	29.5	0.22 (0.82)	−0.20 (0.62)
≥ 41*	12.3	7.0	8.8	20.0	22.7	−0.16 (0.71)	0.01 (0.74)
Mode of delivery^a^							
Natural labour	56.8	55.3	42.2	57.0	55.7	0.29 (0.89)	−0.07 (0.65)
Assisted natural labour	16.0	16.7	30.4	17.2	21.6	0.17 (0.98)	0.13 (0.81)
Caesarean birth	23.5	24.6	23.5	25.0	19.3	0.42 (0.92)	−0.01 (0.74)
Unknown	3.7	3.5	3.9	1.0	3.4	0.36 (0.92)	0.19 (0.74)
With congenital conditions^a^							
Yes	2.5	0.0	0.0	3.0	0.0	−0.96 (0.50)*	0.12 (0.53)
No	97.5	100.0	100.0	97.0	100.0	0.31 (0.92)*	−0.01 (0.71)
Type of feeding^a^							
Never breastfed	56.8	56.1	49.0	53.0	56.8	0.25 (0.81)	−0.02 (0.67)*
Exclusively breastfed 3 months or partially breastfed	28.4	36.0	40.2	39.0	37.5	0.36 (1.09)	0.04 (0.75)*
Exclusively breastfed for 3 months or more	13.6	6.1	7.8	8.0	4.5	0.51 (0.78)	−0.25 (0.80)*
Unknown	1.2	1.8	2.9	0	1.1	−0.03 (0.88)	0.43 (0.52)*
Highest parental education^a^							
≤ 9th grade	33.3	35.1	24.5	32.0	29.5	0.24 (0.92)	−0.09 (0.71)
10th - 11th grade	42.0	42.1	48.0	46.0	45.5	0.36 (0.85)	−0.03 (0.64)
≥ 12th grade	23.5	21.9	24.5	21.0	22.7	0.29 (1.05)	0.12 (0.84)
Unknown	1.2	0.9	2.9	1.0	2.3	0.06 (0.85)	−0.01 (0.77)

^a^%, unless otherwise indicated

*p < 0.05: weight-for-age z-score at birth, weight-for-age z-score at 3 months, weight-for-age z-score at 12 months, and gestational age among different trajectories; growth rates from birth to 3 months between children with and without complete tooth emergence at 12 years old, with or without congenital conditions, and among different gestational ages; and, growth rates from 3 m to 12 months among different gestational ages and type of breast feeding

**Table 2:** Comparison of the socio-demographic bariables and permanent tooth emergence between the included and excluded students

Characteristics	Included (*n* = 485)	Excluded (*n =* 29)	*p*
Gender^a^			0.195
Female	50.3	37.9	
Male	49.7	62.1	
Weight for age z-score; mean (SD)			
Birth	−0.17 (0.91)	−1.77 (1.68)	< 0.001***
3 months	0.10 (0.85)	−0.67 (1.16)	< 0.001***
12 months	0.06 (0.87)	−0.37 (0.96)	0.014
Gestational age (weeks)^a^			–
37	7.8	–	
38	22.3	–	
39	29.9	–	
40	26.2	–	
≥ 41	13.8	–	
Mode of delivery^a^			0.008**
Natural labour	53.2	27.6	
Assisted natural labour	20.4	17.2	
Caesarean birth	23.3	48.3	
Unknown	3.1	6.9	
With congenital conditions^a^			1.000
Yes	99.0	100	
No	1.0	0	
Type of feeding^a^			0.749
Never breastfed	54.2	58.6	
Exclusively breastfed 3 months or partially breastfed	36.5	37.9	
Exclusively breastfed for 3 months or more	7.8	3.4	
Unknown	1.4	0.0	
Highest parental education^a^			0.571
≤ 9th grade	30.9	20.7	
10th - 11th grade	44.7	51.7	
≥ 12th grade	22.7	27.6	
Unknown	1.6	0	
Permanent tooth emergence			
mean (SD)	26.2 (2.5)	25.6 (3.7)	0.350
Complete^a^			
Yes	232	13	0.753
No	253	16	

^a^%, unless otherwise indicated

***p < 0.001

**p < 0.01

Among the 485 students, 232 (47.8%) children had 28 permanent teeth emerged. Second molar had the highest rate of un-emergence (14.2–32.2%), followed by second premolar (5.4–5.6%), canine (0.8–3.3%), first premolar (2.3–2.5%), lateral incisor (0.8–1.4%), central incisor (0–0.8%), and first molar (0.2%), see Table 3. Sixty eight subjects were re-examined to assess the level of examiner reliability throughout the study. The intra-examiners’ intra-class correlation coefficient (ICC) was 0.99 for both examiners and the inter-examiner reliability was 0.97, indicating excellent agreement within and between the examiners.

**Table 3:** Frequency [*n* (%)] of emerged permanent teeth (*n* = 485)

Tooth type	Number (%)	Tooth type	Number (%)
Maxillary		Mandibular	
Central incisor	970 (100.0)	Central incisor	956 (98.6)
Lateral incisor	966 (99.6)	Lateral incisor	951 (98.0)
Canine	902 (94.8)	Canine	959 (98.9)
1st premolar	940 (96.9)	1st premolar	942 (97.1)
2nd premolar	889 (91.6)	2nd premolar	893 (92.1)
1st molar	969 (99.8)	1st molar	969 (99.9)
2nd molar	600 (61.9)	2nd molar	797 (82.2)

Significant association was found between the emergence of permanent tooth and growth rates from birth to 3 months. Unadjusted logistic regression model identified that children with less changes of weight-for-age z-score from birth to 3 months had significantly higher chance of having complete permanent tooth emerged than those with great changes (OR 0.72, 95% CI 0.58, 0.89), Table 5. The significance remained in the adjusted models after adjusting for birth characteristics and socio-demographic factors (OR 0.67, 95% CI 0.54, 0.84) and further adjusted for birth weight (OR 0.67, 95% CI 0.54, 0.84). However, no association was found between emerged permanent teeth and growth trajectories or growth rates from 3 to 12 months in the unadjusted and adjusted models (*p* > 0.05, Tables 4 and 5). Significant association was found between the emergence of permanent tooth and growth rates from birth to 3 months. Unadjusted logistic regression model identified that children with less changes of weight-for-age z-score from birth to 3 months had a significantly higher chance of having complete permanent tooth emerged than those with great changes (OR 0.72, 95% CI 0.58, 0.89), Table 5. The significance remained in the adjusted models after adjusting for birth characteristics and socio-demographic factors (OR 0.67, 95% CI 0.54, 0.84), and further adjusted for birth weight (OR 0.67, 95% CI 0.54, 0.84). However, no association was found between emerged permanent teeth and growth trajectories or growth rates from 3 to 12 months in the unadjusted and adjusted models (*p* > 0.05, Tables 4 and 5).

**Table 4:** Association of growth trajectory at 0–12 months with complete emerged permanent teeth until 12 years of age

Trajectory	Model 1^a^	Model 2^b^	Model 3^c^
	OR (95% CI)	OR (95% CI)	OR (95% CI)
Complete erupted			
I	0.86 (0.48, 1.55)	0.98 (0.52, 1.83)	1.22 (0.63, 2.36)
II	0.85 (0.50, 1.46)	0.95 (0.54, 1.68)	1.20 (0.66, 2.19)
III	1.37 (0.79, 2.39)	1.36 (0.76, 2.44)	1.48 (0.82, 2.66)
IV^d^	1.00	1.00	1.00
V	1.18 (0.67, 2.09)	1.19 (0.65, 2.18)	1.09 (0.60, 1.99)

^a^Model 1: unadjusted

^b^Model 2: adjusted for gestational age (as categorical variable), mode of delivery (natural labour, assisted natural labour, caesarean birth), type of feeding (never breastfed, exclusively breastfed 3 months or partially breastfed, exclusively breastfed for 3 months or more), health status (presence or absence of congenital conditions), highest parental education (≤9th, 10th -11th, ≥ 12th grade), and gender

^c^Model 3: additionally adjusted for z-score for birth weight

^d^Reference category

**Table 5:** Odds ratios for the occurrence of complete emerged permanent teeth until 12 years per unit increases in birth weight z-score and change in weight-for-age z-score at 0–3 months and 3–12 months

	Model 1^a^	Model 2^b^	Model 3^c^
	OR (95% CI)	OR (95% CI)	OR (95% CI)
Birth weight z-score^d,e^			
Complete emerged	1.40 (1.14, 1.72)**	1.45 (1.17, 1.80)**	–
Change of weight-for-age z-score from birth to 3 months^d,f^			
Complete emerged	0.72 (0.58, 0.89)**	0.67 (0.54, 0.84)**	0.67 (0.54, 0.84)**
Change of weight-for-age z-score from 3 months to 12 months^d,e,f^			
Complete emerged	1.23 (0.94, 1.61)	1.23 (0.94, 1.63)	1.31 (0.98, 1.75)

^a^Model 1: unadjusted

^b^Model 2: adjusted for gestational age (as categorical variable), mode of delivery (natural labour, assisted natural labour, caesarean birth), type of feeding (never breastfed, exclusively breastfed 3 months or partially breastfed, exclusively breastfed for 3 months or more), health status (presence or absence of congenital conditions), highest parental education (≤9th, 10th -11th, ≥ 12th grade), and gender

^c^Model 3: additionally adjusted for birth weight for age z-score (or baseline weight for the model looking at growth at 3–12 months)

^d^Association with complete permanent tooth emergence at 12 years of age

^e^One unit change in birth weight z-score is equivalent to about 500 g in girls and 600 g in boys

^f^One unit change in weight z-score at 0–3 months and 3–12 months is equivalent to the distance between adjacent centile lines or 2nd centile, 50th centile, 84th centile, and 98th centile on standard growth charts

**p < 0.01

Results from the tooth level in bivariate and multivariate analyses showed that students who had a slow growth rate from birth to 3 months of age had a higher chance having the maxillary second molars erupted at their 12 years of age (Tables 6 and 7).

**Table 6:** Associations between the emergence of each tooth type and growth rates from birth to 3 months

Tooth type	Mean Growth rate from birth to 3 months^a^ (SD)				*p*
	Complete emergence		Incomplete emergence		
Maxillary					
Central incisor	0.25	(0.89)	–	–	–
Lateral incisor	0.25	(0.89)	0.29	(0.57)	0.917
Canine	0.23	(0.88)	0.51	(1.01)	0.083
1st premolar	0.25	(0.89)	0.24	(0.97)	0.981
2nd premolar	0.24	(0.89)	0.28	(0.91)	0.799
1st molar	0.25	(0.89)	0.69	–	0.618
2nd molar	0.17	(0.91)	0.35	(0.85)	0.029*
Mandibular					
Central incisor	0.25	(0.89)	0.20	(0.75)	0.882
Lateral incisor	0.25	(0.89)	0.32	(0.67)	0.780
Canine	0.25	(0.89)	0.13	(1.12)	0.737
1st premolar	0.24	(0.89)	0.36	(0.73)	0.596
2nd premolar	0.25	(0.90)	0.19	(0.77)	0.621
1st molar	0.25	(0.89)	0.06	–	0.831
2nd molar	0.22	(0.90)	0.34	(0.83)	0.256

^a^Growth rate from birth to 3 months = weight *z*-score – birth weight *z*-score

Two sample *t*-test; *p < 0.05

**Table 7:** Odds Ratios for the Occurrence of Complete Emerged Maxillary Second Molar until 12 Years Per Unit Increases in Birth Weight z-score and Change in Weight-for-age z-score at 0–3 Months and 3–12 Months

	Model 1^a^	Model 2^b^	Model 3^c^
	OR (95% CI)	OR (95% CI)	OR (95% CI)
Birth weight z-score^d,e^			
Complete emerged	1.25 (1.02, 1.52)*	1.29 (1.05, 1.59)*	–
Change of weight-for-age z-score from birth to 3 months^d,f^			
Complete emerged	0.79 (0.64, 0.98)*	0.75 (0.60, 0.94)*	0.75 (0.60, 0.94)*
Change of weight-for-age z-score from 3 months to 12 months^d,e,f^			
Complete emerged	1.20 (0.91, 1.58)	1.22 (0.92, 1.61)	1.26 (0.95, 1.68)

^a^Model 1: unadjusted

^b^Model 2: adjusted for gestational age (as categorical variable), mode of delivery (natural labour, assisted natural labour, caesarean birth), type of feeding (never breastfed, exclusively breastfed 3 months or partially breastfed, exclusively breastfed for 3 months or more), health status (presence or absence of congenital conditions), highest parental education (≤9th, 10th -11th, ≥ 12th grade), and gender

^c^Model 3: additionally adjusted for birth weight for age z-score (or baseline weight for the model looking at growth at 3–12 months)

^d^Association with complete permanent tooth emergence at 12 years of age

^e^One unit change in birth weight z-score is equivalent to about 500 g in girls and 600 g in boys

^f^One unit change in weight z-score at 0–3 months and 3–12 months is equivalent to the distance between adjacent centile lines or 2nd centile, 50th centile, 84th centile, and 98th centile on standard growth charts

*p < 0.05

## Discussion

There is a growing interest in seeking specific factors to recognize trends in oral health over the lifespan of individuals and to determine whether such factors can alter subsequent oral health events through life-course studies. The investigation of health status during the life-course helps to understand and evaluate the effects of genetic and environmental factors occurred at different ages of life on the occurrence of several health related events [26]. It is universally accepted that birth weight is a substitute of nutritional status in utero, and weight gain is a surrogate of infant growth [7]. In our study, birth weight was used to represent the nutritional status in utero*,* while two growth parameters were employed to represent the nutritional status during infancy. We provided information and conducted statistical analyses on i) five growth trajectories during the first year of life which was considered as categorical variables; and ii) two growth rates (changes of weight-for-age *z*-scores from birth to 3 months and from 3 months to 12 months) which were treated as continuous variables.

To the best of our knowledge, this is the first study to investigate the relationship between infant growth and subsequent emergence of permanent tooth at 12 years of age considering growth trajectories and growth rates in a sub-sample from a Chinese birth cohort. The study comprised of 485 students from a birth cohort with an approximately equal distribution of gender (241 boys and 244 girls). It was our observation that children of a higher birth weight had a significant higher chance of having complete emergence of permanent teeth in the permanent dentition at their 12 years of age; and a slow growth rate from birth to 3 months of age had a significant higher chance of having complete emergence of permanent teeth in permanent dentition at their 12 years of age. The results should be treated with caution because this article described statistical associations between birth weight development and tooth emergence in a cross-sectional sample. Using cross-sectional design with a one-time measurement on a phenomenon like growth and tooth emergence could not yield a cause-effect conclusion. However, it provided hints for further studies, in terms of studies with multiple age groups, longitudinal studies, as well as experimental studies. Moreover, the dichotomous analysis of tooth emergence status limited detailed exploration of individual tooth eruption though it is methodologically very difficult to collect data of the exact emergence time.

The finding of association between higher birth weight and complete emergence of permanent teeth appears to be similar to reports by other investigators that subjects with low birth weight and/or preterm birth had a higher chance of delayed tooth eruption compared to those with the normal birth weight and/or full term birth [27–30]. It was demonstrated in the literature that there was only an association between birth weight and primary tooth eruption because these studies mainly focused on the primary dentition. From our results it is suggested that the effect of birth weight extends far beyond the initial stage of tooth emergence and persists into the whole process. Growth parameters in the early months of life when growth rate is fastest may affect the teething time.

Interestingly it was found in this study that slow infant growth in the first three months of life was related to the complete emergence of permanent teeth at 12 years of age. The results cannot be fully explained by current knowledge because no study has sought to investigate the possible relationship between infant growth and emergence of permanent teeth in 12-year-old children; this also precludes the possibility of comparing our findings with other published data. However, other investigations in Medicine showed that rapid infant growth are related to certain health problems [31, 32]. According to the DOHaD (Developmental Origins of Health and Disease) hypothesizes, disorders later in life originate through unbalanced nutrition during infancy [33]. Furthermore, optimal growth is of great importance during infancy [34]. A smooth and slow growth within the “normal” range growth in the first three months of life might be important to balance the metabolic load and capacity in this “critical period” which considered having influences on health events later in life [31]. The exact mechanism is worth of further investigations.

When the association of birth weight and growth rates during the first three months of life on permanent tooth emergence were further investigated for each tooth type (Table 6), significant association was found only for maxillary second molars. The significance was further confirmed by logistic regressions (Table 7). This suggests that the overall association of birth weight and growth rates during the first three months of life on the status of tooth emergence at 12 years old is mostly attributable to maxillary second molars. It is widely accepted that the age of 12 years is within the normal range for the emergence of 28 permanent teeth and the teeth erupt in a particular sequence. With this knowledge in mind it is not difficult to explain the finding of the significant association because the maxillary second molars are usually the last in the sequence to erupt.

The lack of an association between growth trajectories and permanent tooth emergence in secondary school children, found in this study, may have been because the sub-sample was drawn from a randomized stratified sampling procedure. Only term births (gestation ≥37 weeks) were included to generate the growth trajectories in the study population because different trajectories have been found for term births and preterm births [25]. This is the reason why there were differences in the mode of delivery, birth weight for age z-score and 3 months weight for age z-score between included and excluded participants (Table 2). Furthermore, the categorization of the growth trajectories was based on the growth during the first year of life instead of during the first three months of life. Thus, the finding of the study may be compromised by the lack of preterm birth participants which is a significant marker of inadequate nutrition during fetal development and “prolonged” growth trajectories. The results of this study cannot be generalised to the population. In addition, the present study was school based where radiographic examination and dental history were unavailable to determine if the un-emergence of teeth was due to hypodontia or extraction. However, the prevalence figures of hypodontic second molars and extractions due to caries/orthodontic treatment were reported to be nearly 0% [35, 36]. Therefore, the results of this study are unlikely to be affected significantly by this limitation. Finally, only body weight was measured and analysed in this study. Although body weight, body length/height and head circumference are measurements recommended by WHO to monitor infant growth, body weight provide more reasonably valid and precise readings as it is measured by mechanical and electronic scales. Body length/height provides less precisely measure because of variations in posture and muscle tone among infants while infants lie on the measuring table. Head circumference provides better reproducible results than infant body length/height; however, the presence of head molding at birth may affect the measurement [37]. Other anthropometric measurements and local factors which might have an impact on tooth eruption, for example, peripheral adiposity, ankylosis or early loss of a primary tooth, impaction, crowding, and dental caries experience, were not discussed in this paper. These potential factors, which have infrequently been considered in the literature [38], either, may be worthy of further investigation to support or refute claims.

Further investigations, especially longitudinal studies to monitor growth changes such as infant’s growth in length, and exact timing of individual tooth emergence, as well as experimental studies on the molecular mechanisms triggering dental development are required to expand our knowledge in tooth eruption/emergence.

## Conclusions

In summary, the results of the logistic regression analyses indicated that infants in a Chinese birth cohort with a heavy birth weight and slow growth during the first three months of life were more likely to have complete emergence of permanent teeth at 12 years of age. The first three months of life might be a critical period for the permanent tooth emergence later in life, while slow growth during this period of time might have beneficial effects on tooth emergence. These findings may have clinical significance of predicting dental events later in life in terms of the risk for dental caries due to prolonged exposure in the oral cavity, and the probability of malocclusions due to unfavorable eruption sequence. Other applications include medical/legal issues and forensic investigations.

## Abbreviations

DOHaD: Developmental Origins of Health and Disease
LED: light-emitting diode
MCHCs: Maternal and Child Health Centers
WHO: World Health Organization
